# Students' Nutrition Literacy and the Existence of Health Care Providers in Iranian Schools

**DOI:** 10.34172/jrhs.2020.11

**Published:** 2020-05-05

**Authors:** Ahmad Mehri, Nasrin Jafari, Isa Akbarzadeh, Fatemeh Hadavand Siri, Nategh Abbassgholizadeh

**Affiliations:** ^1^Department of Epidemiology and Biostatistics, School of Public Health, Tehran University of Medical Sciences, Tehran, Iran; ^2^Department of Public Health, School of Health, Student Research Committee, Ardebil University of Medical Sciences, Ardebil, Iran; ^3^Department of Epidemiology, School of Public Health and Safety, Shahid Beheshti University of Medical Sciences, Tehran, Iran; ^4^Department of public health, School of Health, Ardebil University of medical sciences, Ardebil, Iran,

**Keywords:** Nutrition surveys, Health literacy, School nursing, Student health services

## Abstract

**Background:** The objective of this study was to investigate the relationship between Students' nutrition literacy and the existence of health care providers in Iranian schools.

**Study design:** A cross-sectional study.

**Methods:** This study was conducted on 504 students in Ardebil City, northwestern Iran from Oct 2017 to Jan 2018. The FLINT questionnaire was used to assessment the food and nutrition literacy. Socio-demographic characteristics and the existence of health care providers were collected using demographic questionnaire.

**Results:** Nearly 75% of students had not a health care provider. Most students had a low FNLIT (62% males and 58.1% females). The probability of low FNLIT was lower in students with health care providers than those without them (OR=0.46, CI 95%; 0.10, 0.91).

**Conclusion:** One of the reasons for the low nutritional literacy of students may be due to the lack of health care providers in schools. Health educational administrators employ specialized health care providers in Iranian schools.

## Introduction

The UNESCO has set one of its goals in planning elementary education, improving the growth and development of potential talents, and establishing proper health behaviors in children^1,2^. These behaviors can provide the foundation for a healthy lifestyle ^3^. In Iran, some factors may endanger the health of students. The prevalence of obesity and metabolic syndrome is high in Iranian adolescents^4^, and students' knowledge of different aspects of nutrition is also inadequate ^5^. Besides, the prevalence of overweight in the world is 5.6% and in Iran is between 5% and 10%^6,7^, which may increase in the future and threaten future adolescent health. Moreover, 73% of adolescents aged 14-18 yr were consuming fruits and vegetables below the recommended guideline amounts and consumed high amounts of fatty foods. On the other hand, more than 50% of them had fewer than 1.5 servings of fruit and vegetables daily, and 41% consume less than one serving/d ^8^.

One of the essential aspects of literacy that affects student's health is food and nutrition literacy (FNLT). FNLT is a degree of ability for individuals to acquire, process, understand information and skills necessary to make appropriate nutrition decisions ^9^. Studies suggested a positive association between food literacy and adolescents’ dietary intake^10,11^ and adolescents with better food knowledge, and frequent food preparation behaviors were shown to have healthier dietary practices^12^.

The existence of health care providers in schools can affect students' nutritional literacy and health^13^. A school health nurse performs school health activities in many countries that this role was first started by Lilian Wald in the US and gradually expanded^14^. The health care provider in schools is tasked with educating and providing health, nutrition, and psychological counseling to enhance literacy, enhance self-care, and improve health behaviors^15,16^. The existence of health care providers in schools has a significant positive impact on health-related behaviors, especially on elementary school students ^17-19^. For example, for dental and oral hygiene, studies claimed that the existence of health care providers in schools causes health behaviors improvement^20-22^. Iranian Parliament's laws have required the Ministry of Education to apply health care in all schools ^23^. According to the Ministry of Education's job classification, graduates with hygiene, nutrition, nursing, and midwifery degrees can work in schools as health care providers^14^. Despite legal requirements, many schools in Iran still lack health care providers with academic health sciences degree. On the other hand, students in schools that have health care providers are subject to specialized health monitoring and evaluation, and in schools without specialized health care, students may not be considered for health concepts such as nutrition and health literacy ^24^.

However, based on our knowledge, there is no study of the relationship between student nutrition literacy and the existence of health care providers to produce the necessary scientific document for health and education policymakers in Iran. We aimed to assess the relationship between nutrition literacy and the existence of school health care in Iranian schools.

## Methods

### Study Design and Participants

This cross-sectional study was conducted from Oct 2017 to Jan 2018 and included 504, 13-15 yr students in Ardebil, a city placed in the northwest of Iran. The General Office of Education in Ardebil groups educational districts into two groups based on socioeconomic status; District One (north of Ardebil) and District two (south of Ardebil). The recruited students were selected from each of these two groups. A weighted sampling was conducted from six clusters (three from every two educational districts), according to their student’s population density. Then a probability proportional to the size of the target population method was used to select governmental and non-governmental schools from these six districts. Afterward, an equal number of students from 13, 14, and 15 yr were randomly selected from each school.

These selected students and their parents were invited to participate in the study by a written invitation and information card. However, at the beginning of the study, verbal informed consent was obtained from students and their parents.

### Food and nutrition literacy assessment

The FLINT questionnaire was used for food and nutrition literacy assessments. This tool contains 46 items assessing FLINT in two specific cognition and skills domains. The cognition domain consists of two subscales, including understanding food and nutrition information, and nutritional health knowledge. The skills domain consists of seven subscales and evaluates examining functional FNLIT, interactive FNLIT, food choice literacy, critical FNLIT, and food label literacy (Table 1 lists some questions from each subscale). This self-administrated questionnaire was completed by students on their own and approved by their parents. Parental confirmation was that for some demographic questions, which might not be sufficient information for students. It would then be completed and approved by the parents. Parents were not involved in completing the nutrition literacy questions. If the student did not understand each question, it was explained by the interviewer. The whole process of completing the questionnaire was under the supervision of the interviewer. Studies evaluating the validity and reliability of this tool indicated that the scale items were generally easy to read and comprehend for students^25^. The scores of questions in each domain were summed to calculate the score of each domain and subscales. Students nutrition literacy were classified into three categories: low FNLIT (≤51), moderate FNLIT (>51– <74) and high FNLIT (≥74) ^26^.

**Table 1:** List of some questions in each subscale

**Domain /subscale**	**Questions**
**Cognition**
understanding food and nutrition information	I can easily understand the nutritional content that is printed in magazines and brochures. When shopping for food, the date of manufacture and expiration on the packaging is essential to me, and I can understand it.
Nutritional health knowledge	Salty snacks such as chips and muffins are harmful to health. Consuming sausage and fast food increases the risk of cancer.
**Skill**
functional FNLIT	If I have questions about nutrition, I ask my parents or my teacher. I wash and prepare the fruits and vegetables myself.
Interactive FNLIT	I can and will not resist fast food and fatty foods. I can easily say no to my friends' suggestions for unhealthy food.
Critical FNLIT	I usually try new foods I haven't eaten yet. I can, for my pocket money, buy foods from the buffet that are useful.
Food choice literacy	Have you ever seen the nutritional information table on food packaging? Do you choose your food based on the information on the packaging?
Food label literacy	Is the red color on the nutrition information of any food packaging above the limit? If the amount of salt in the food information in the food packaging is green, what is the limit?

### Baseline characteristics

Baseline characteristics including age, family size (<4, 4 and >4), father’s age tertile (T1: 30–45, T2: 45–60 and T3: ≥60 yr old), mother’s age tertile (T1: 30–45, T2: 45–60 and ≥ 60 yr old), parents’ education (illiterate or ≤5yr, 6–9 yr or diploma and associate degree or higher), father’s job status (worker, employee, self-manager, unemployed), mother’s occupation status (housewife and work outside the home), house ownership status (owner, tenant), and school type (governmental, non-governmental), age (13, 14, and 15) and the existence health care provider (yes, no) were collected by questionnaire alongside with interviewing with students and approval of their mother.

### Statistical analysis methods

At each school, the health care providers do not cover all students. Some school classes have a health care provider, and some do not. For this reason, a comparison of student literacy between students with and without health care provider was performed at the individual level. Mean, and the standard deviation were reported for describing quantitative variables with normal distribution and counts and percentages for categorical variables. A Chi-square test was used to analyze the differences in baseline characteristics and FNLT between girls and boys. A logistic regression model was fit to measure the effect size of the independent variables on FLINT (dependent variable) prediction. All analyses were performed by Stata ver. 14.0.

### Ethical considerations

The Ethics Committee of Ardebil University of Medical Sciences approved this study with the ethics code of IR.ARUMS.VCR.REC.1398.012. Verbal consent was obtained from students to participate in the study. Parents also verbally consented their child to participate in the study. The confidentiality of the obtained data was also guaranteed.

## Results

### Demographic and socioeconomic characteristics of the study participants

The baseline characteristics of the sample are summarized in Table 2. Overall, 504 students (253 boys and 251 girls) participated in the study. The mean age was 13.77 ±0.71 years. Nearly two-thirds of students have not health care provider (75%). The girls and boys significantly differ in some demographic and parental related factors, including mother age (*P=* 0.021), family size (*P* =0.038), father’s education (*P* =0.043), and existing health care provider (*P* =0.001). About 69% of schools in boys and 76.5% of schools in girls were governmental.

**Table 2:** Baseline characteristics of the participants (n=504)

**Variables**	**Boys, n=253**		**Girls, N=251**		**Total, n=504**		***P-*** **value**
	**Number**	**Percent**	**Number**	**Percent**	**Number**	**Percent**	
**Age (yr)**							0.421
13	96	37.9	102	40.6	198	39.3	
14	121	47.8	101	40.2	222	44.0	
15	36	14.2	48	19.2	84	16.7	
**School type**							0.216
Governmental	174	68.8	192	76.5	366	72.6	
Non-government	79	31.2	59	23.5	138	27.4	
**Father age (yr)**							0.132
30-45	147	58.1	167	66.5	314	62.3	
45-60	94	37.2	81	32.3	175	34.7	
≥60	12	4.7	3	1.2	15	3.0	
**Mother Age (yr)**							0.021
30-45	14	5.8	7	2.8	21	4.3	
45-60	199	82.2	227	90.8	426	86.6	
≥60	29	12.0	16	6.4	45	9.1	
**Family size**							0.038
<4	236	93.3	243	96.8	479	95.0	
4	8	3.2	7	2.8	15	3.0	
>4	9	3.6	1	0.4	10	2.0	
**Father’s job**							0.614
Employee/clerk	88	34.8	75	29.9	163	32.3	
Worker	33	13.0	35	13.9	68	13.5	
Self-manager	123	48.6	134	53.4	257	51.0	
Unemployed	9	3.6	7	2.8	16	3.2	
**Mother employment**							0.481
working	23	9.1	24	9.6	47	9.3	
Housewife	230	90.9	227	90.4	457	90.7	
**House ownership status**							0.341
Owner	222	87.7	217	86.5	439	87.1	
Tenant	31	12.3	34	13.5	65	12.9	
**Father’s education years**							0.043
≤5	86	34.0	89	35.5	175	34.7	
6-12	74	29.2	94	37.5	168	33.3	
Academic	93	36.8	68	27.1	161	31.9	
**Mother’s education years**							0.301
≤5	98	38.7	98	39.0	196	38.9	
6-12	86	34.0	98	39.0	184	36.5	
Academic	69	27.3	55	22.0	124	24.6	
**Existence Health care provider**							0.001
No	190	75.1	122	48.6	312	61.9	
Yes	63	24.9	129	51.4	192	38.1	

### Total food and nutrition literacy (FNLIT) and its subscales characteristics:

Total FNLIT in two domains (cognitive and skills) and nine subscales are reported in Table 3. Most students had a low FNLIT (62% in boys and 58.1% in girls). In terms of the FNLIT cognitive domain, the high proportion of students also had moderate status (61% in boys and 65.3% in girls). Moreover, most students were at a low level in Nutritional health knowledge (87.7% in boys and 86.1% in girls), and only 1.6% of all students had a high level of FNLIT skill. Most students had a high level in Food Choice Literacy (75.4%), but a high proportion of students had a low level in Food label literacy (66.4% in boys and 59.8% in girls). Gender differences were significant in Understanding food and nutrition info, Functional FNLIT, and Interactive FNLIT subscales (*P* <0.05).

**Table 3:** The status of FNLIT in 13–15-year-old participants (n=504)

**FNLIT and its subscales**	**Boys, n=253**		**Girls, n=251**		**Total, n=504**		***P*** **-value**
	**Number**	**Percent**	**Number**	**Percent**	**Number**	**Percent**	
**Total FNLIT**							0.902
Low	157	62.0	146	58.1	303	60.0	
Moderate	61	24.1	62	24.7	123	24.4	
High	35	13.8	43	17.2	78	15.6	
**FNLIT cognitive domain**							0.081
Low	83	33.0	70	27.1	153	30.1	
Moderate	153	61.0	164	65.3	317	63.1	
High	15	6.0	19	7.6	34	6.8	
**FNLIT cognitive domain subscales**							
**Understanding food and nutrition info**							0.012
Low	55	21.7	79	31.5	134	26.6	
Moderate	177	70.0	162	64.5	339	67.3	
High	21	8.3	10	4.0	31	6.2	
**Nutritional health knowledge**							0.842
Low	222	87.7	216	86.0	438	86.9	
Moderate	26	10.3	30	12.0	56	11.1	
High	5	2.0	5	2.0	10	2.0	
**FNLIT skill domain**							0.331
Low	136	53.8	116	46.2	252	50.0	
Moderate	23	9.1	44	17.5	67	13.3	
High	94	37.2	91	96.3	185	36.7	
**FNLIT skill domain subscales**							
**Functional FNLIT**							0.014
Low	61	24.1	42	16.7	103	20.4	
Moderate	187	73.9	206	82.1	393	78.0	
High	5	2.0	3	1.2	8	1.6	
**Interactive FNLIT**							0.001
Low	43	17.0	85	33.9	128	25.4	
Moderate	136	53.8	107	42.6	243	48.2	
High	74	29.2	59	23.5	133	26.4	
**Critical FNLIT**							0.241
Low	34	13.4	35	13.9	69	13.7	
Moderate	79	31.2	96	38.2	175	34.7	
High	140	55.3	120	47.8	206	51.6	
**Food choice literacy**							0.874
Low	17	6.7	15	6.0	32	6.3	
Moderate	48	19.0	44	17.5	92	18.3	
High	148	74.3	192	76.5	380	75.4	
**Food label literacy**							0.011
Low	168	66.4	150	59.8	318	63.1	
Moderate	67	26.5	92	36.7	159	31.5	
High	18	7.1	9	3.6	27	5.4	

### FNLIT and existence health care provider:

In Table 4, multiple logistic regression was used to assess the relationship between the low FNLIT and its domains and subscales and existence health care provider adjusted to other demographic variables. Moderate and high levels were considered as a group. Overall, the probability of low FNLIT was lower in students with health care provider than those without health care provider (OR=0.46, CI 95%= 0.1 to 0.91). In addition, odds of low FNLIT adjust for other variables was lower in student with Health care provider than without health care provider in nutritional health knowledge (OR=0.61, CI 95%= 0.29 to 0.83), functional literacy (OR=0.32, CI 95%= 0.11 to 0.79), food choice literacy (OR=0.89, CI 95%= 0.13 to 0.98) and critical literacy subscales (OR=0.24, CI 95% =0.09 to 0.63). There was no relationship between the existence of health care providers and food labeling literacy, interactive literacy, and understanding food and nutrition info subscales.

**Table 4:** Adjusted odds ratios (95% CI) of FNLIT scale and subscale for socioeconomic factors of 13–15 year old students in Ardebil (n=504)

**Variables**	**Total scale**			**Cognitive domain**			**Skill domain**				
	**Low FNLIT**		**Low** **understanding** **food and** **nutrition info**	**Low nutritional** **health knowledge**		**Low functional** **literacy**	**Low food** **choice literacy**	**Low interactive** **literacy**	**Low critical** **literacy**	**Low food** **labeling literacy**
Gender										
Boy	1.00		1.00	1.00		1.00	1.00	1.00	1.00	1.00
girl	0.60 (0.24, 0.72)		2.00 (0.87, 4.61)	1.10 (0.66, 2.01)		0.44 (0.24, 0.78)	1.07 (0.47, 2.36)	0.37 (0.23, 0.58)	1.03 (0.60, 2.64)	1.41 (0.97, 2.23)
School type										
Governmental	1.00		1.00	1.00		1.00	1.00	1.00	1.00	1.00
Non-government	0.80 (0.32, 0.85)		1.02 (0.37, 2.82)	0.47 (0.22, 0.85)		1.52 (0.47, 3.22)	3.74 (1.11, 12.60)	0.98 (0.95, 1.67)	1.48 (0.75, 2.95)	0.82 (0.52, 1.36)
Father’s job										
Employee/clerk	1.00		1.00	1.00		1.00	1.00	1.00	1.00	1.00
Worker	5.91 (0.94, 6.7)		0.76 (0.17, 3.34)	1.50 (0.43, 2.12)		1.53 (0.47, 4.31)	1.09 (0.25, 4.81)	0.52 (0.19, 1.36)	0.66 (0.22, 1.93)	0.79 (0.37, 1.71)
Self-manager	3.64 (1.43, 4.92)		0.90 (0.29, 2.71)	0.53 (0.21,1.87)		2.32 (0.86, 2.63)	1.13 (0.35, 3.46)	1.33 (0.73, 2.49)	0.65 (0.31, 3.36)	0.83 (0.48, 1.44)
Unemployed	2.77 (1.64, 3.51)		1.03 (0.17, 2.48)	0.31 (0.12, 1.14)		3.04 (1.03, 4.27)	3.36 (1.02, 4.93)	0.75 (0.14, 3.92)	2.36 (1.23, 3.02)	1.01 (0.32, 3.74)
Mother employment										
working	1.00		1.00	1.00		1.00	1.00	1.00	1.00	1.00
Housewife	0.36 (0.12, 0.60)		1.41 (0.25, 4.34)	0.24 (0.06, 0.93)		1.18 (0.41, 3.50)	0.88 (0.19, 4.41)	1.21 (0.49, 2.92)	0.66 (0.21, 0.74)	0.44 (0.02, 0.87)
House ownership status										
Owner	1.00		1.00	1.00		1.00	1.00	1.00	1.00	1.00
Tenant	2.67 (0.45, 3.9)		1.34 (0.36, 3.26)	1.08 (0.48, 2.44)		1.54 (0.62, 3.85)	1.36 (0.37, 4.96)	1.68 (0.82, 3.47)	1.58 (0.64, 2.39)	1.43 (0.80, 2.52)
Father’s education years										
0-5	1.00		1.00	1.00		1.00	1.00	1.00	1.00	1.00
6-12	5.94 (0.74, 8.12)		0.76 (0.24, 2.34)	0.71 (0.23, 2.27)		1.07 (0.31, 2.31)	0.31 (0.10, 1.95)	0.80 (0.32, 2.63)	0.75 (0.35, 1.63)	1.19 (0.63, 1.88)
Academic	0.37 (0.12, 0.71)		0.92 (0.17, 3.10)	0.52 (0.34, 1.68)		1.05 (0.87, 1.62)	0.39 (0.01, 0.98)	0.79 (0.41, 3.63)	0.40 (0.14, 1.17)	1.16 (0.54, 2.23)
Mother’s education years										
0-5	1.00		1.00	1.00		1.00	1.00	1.00	1.00	1.00
6-12	0.64 (0.24, 2.81)		0.24 (0.37, 2.47)	1.63 (0.78, 2.24)		1.05 (0.49, 2.48)	0.36 (0.21, 0.79)	1.66 (0.64, 2.69)	1.95 (0.68, 2.91)	1.33 (0.77, 2.03)
Academic	0.38 (0.13, 0.76)		0.67 (0.24, 0.93)	0.71 (0.23, 0.84)		0.65 (0.21, 0.82)	0.42 (0.21, 0.78)	2.15 (0.71, 3.63)	0.32 (0.13, 0.64)	0.58 (0.21, 0.69)
Existence health care provider										
No	1.00		1.00	1.00		1.00	1.00	1.00	1.00	1.00
Yes	0.46 (0.12, 0.91)		0.81 (0.36, 2.34)	0.61 (0.29, 0.83)		0.32 (0.11, 0.79)	0.89 (0.13, 0.98)	1.32 (0.83, 2.90)	0.24 (0.09, 0.63)	0.60 (0.21, 2.67)

## Discussion

We aimed to investigate the relationship between students' nutritional literacy and the existence of health care providers in Iranian schools. Our results showed that six out of every ten students had low FNLIT, and about quarter students had moderate FNLIT. In our study, the difference in FNLIT between cognitive and skill domains was also examined, and as the results showed, third of the students had low FNLIT in the cognitive domain, and in the skill domain, this index increased to 50%. Similar to our results, in students of Tehran (capital of Iran), skill FNLIT was lower than cognitive FNLIT^26^. These results appear to be one of the crucial problems in the content of education in Iranian schools that focus more on theoretical content rather than practical skill-based topics.

A study by analyzing the educational content available in Iranian schools, ^27^ have also shown that more focus was on theoretical content rather than practical skill-based topics. Although FNLIT alone is not enough factor in improving health and nutritional behaviors in students, practical education and increasing FNLIT should be considered.

Our results showed that students with health care provider had better nutritional literacy than students without health care provider. This difference was also observed in nutritional health knowledge, functional literacy, food choice literacy and critical literacy subscales. There are limited studies of the relationship between the existence of health care providers and nutritional literacy. The existence of a school health nurse as a school health provider was important for enhancing health literacy and nutrition interventions ^28^. Another study ^29^ has shown the need for specialized health care providers to implement programs that promote proper nutritional behaviors and oral health. Other similar studies have also shown a relationship between the existence of specialized health care providers in schools and rising in students' health and nutrition aspects ^30-33^. Increasing the health literacy of school health care provider had a positive impact on students' health literacy ^34^. Since student nutrition is a proxy for health, it can affect their nutrition literacy. Although our study only examined students' nutritional literacy, a study of 300 students in Tehran showed that students with better health behaviors had better health behaviors regarding physical activity and oral health ^24^. The existence of health care providers in Iranian schools can play an essential role in improving the student’s health and nutritional literacy.In terms of the limitations of this study, the information was measured as a self-report. It can lead to under-reporting or over-reporting. Although there are many social, cultural, and economic factors affecting students' nutrition literacy, only some of these factors were investigated in this study, which requires further studies to investigate other factors.

## Conclusion

Although the relationship between the existence of health care providers in schools and the improvement of various aspects of health in students has been partially confirmed, the existence of this specialized force has not been addressed in iranian schools yet. Overall, 75% of students in Ardebil city did not have health care. One of the reasons for the low nutritional literacy of students, especially in the skill domain, maybe due to the lack of health care providers in schools. Health policymakers and educational administrators employ specialized health care providers in schools to improve student health and implement specialized health care and self-care education interventions.

## Acknowledgements

This cross-sectional study was support by Ardebil University of Medical Sciences.

## Conflict of interest

None.

## Funding

None.

## Highlights

- Nearly 75% of students had not health care provider.
- Most students had low food and nutrition literacy (62% males and 58.1% females).
- Moreover, most students were at a low level of nutritional health knowledge (87.7% males and 86.1% females).
- Probability of low food and nutrition literacy was lower in students with health care providers than those without them.
